# Pharmacological principles of intravitreal drug therapy and their implications for clinical practice: a primer for the ophthalmologist

**DOI:** 10.1038/s41433-026-04540-y

**Published:** 2026-05-21

**Authors:** Samia Ezzine, Richard Gale, Clare Bailey, Praveen J. Patel, Sobha Sivaprasad, Zinab Keshk, Thomas Eissing, Joachim Höchel, Rose Gilbert, Peter Morgan-Warren

**Affiliations:** 1 Bayer Inc, Montréal, QC Canada; 2 Eye Clinic, York and Scarborough Teaching Hospitals NHS Foundation Trust; Hull York Medical School, University of York, York, UK; 3 Eye Clinic, Bristol Eye Hospital, University Hospitals Bristol and Weston NHS Foundation Trust, Bristol, UK; 4 NIHR Moorfields Biomedical Research Centre at Moorfields Eye Hospital NHS Foundation Trust and UCL Institute of Ophthalmology, London, UK; 5 Bayer plc, Reading, UK; 6 Bayer AG—Pharmaceuticals Division, Leverkusen, Germany; 7 Bayer AG—Pharmaceuticals Division, Berlin, Germany; 8 Bayer Consumer Care AG, Basel, Switzerland

**Keywords:** Pharmacodynamics, Pharmacokinetics

## Abstract

Intravitreal (IVT) anti–vascular endothelial growth factor (anti-VEGF) therapies are the standard of care for retinal diseases such as neovascular age-related macular degeneration (nAMD) and diabetic macular oedema (DMO). Despite the favourable efficacy and safety profiles of these therapies, decreasing the treatment burden is still an unmet need because frequent injections may be required over many years. The pharmacokinetic and pharmacodynamic properties of IVT therapies, such as ocular half-life and clearance, can affect the duration of VEGF suppression and thus influence clinical outcomes. Although some properties are inherent to the drug molecule (such as molecular weight, binding affinity and potency) and cannot be altered without changing the structure of the molecule, other factors (such as the dose of the drug) can be increased, which may prolong VEGF suppression time in the eye and, in turn, may lead to a more durable effect of the drug. In addition to pharmacokinetic and pharmacodynamic properties, individual patient factors such as age, surgical history and disease status can also affect the pharmacokinetics, pharmacodynamics and observed effectiveness of a drug. This article reviews the key pharmacological properties of IVT anti-VEGF treatments for nAMD and DMO often referred to in the literature, and aims to elucidate their meaning and clinical relevance for managing retinal diseases.

## Introduction

In the past two decades, the treatment landscape for neovascular age-related macular degeneration (nAMD) and diabetic macular oedema (DMO) has been revolutionised by intravitreal (IVT) anti–vascular endothelial growth factor (anti-VEGF) therapies, such as ranibizumab, aflibercept, brolucizumab, faricimab and bevacizumab gamma. When compared with sham injections or previous standards of therapy, such as laser therapy for DMO and photodynamic therapy for certain subtypes of nAMD, these drugs have demonstrated improved outcomes in clinical trials in patients with these conditions, helping to prevent vision loss, along with favourable tolerability and safety profiles [1–3]. As such, these drugs are now the standard of care for the treatment of nAMD and DMO as well as several other indications affecting retinal vascularisation.

However, unmet needs remain with IVT anti-VEGF therapies and their dosing regimens, which have been established over the last two decades (see Table 1 [4–9]). Anti-VEGF therapies often require administration at frequent intervals to maintain improvements in vision, which can result in a high treatment burden on patients, caregivers and the healthcare system [10, 11]. This includes feelings of anxiety related to injections; time spent travelling to, waiting for and recovering from treatment; travel-related costs; and patients having to ask caregivers for support attending treatment visits [11–13]. The need for frequent injections also places a burden on ophthalmology clinics. In the UK, ophthalmology clinics can be the busiest outpatient speciality, and the increased need for treatment results in patient backlogs and delays in receiving treatment, which in turn can put patients’ safety and vision at risk [14, 15].

**Table 1:** Overview of available IVT anti-VEGF agents for diseases affecting retinal vascularisation, including nAMD and DMO [4–9].

IVT anti-VEGF agent	Ranibizumab [4]	Aflibercept [5]	Brolucizumab [6]	Faricimab [7]	Bevacizumab gamma [8]
**2D representation of the molecular structure**^**a**^					
**Therapeutic indications (in Europe)**^**b**^	nAMD, DMO, PDR, CNV, macular oedema secondary to RVO, and ROP with zone I, zone II or AP-ROP	nAMD, DMO, mCNV, macular oedema secondary to RVO, and ROP with zone I, zone II or AP-ROP	nAMD and DMO	nAMD, DMO and macular oedema secondary to RVO	nAMD
**Licensed dose(s)**^**b**^	0.5 mg / 0.05 ml (nAMD, DMO, PDR, CNV, macular oedema secondary to RVO) 0.2 mg / 0.02 ml^c^ (ROP)	2 mg / 0.05 ml (nAMD, DMO, mCNV, macular oedema secondary to RVO) 8 mg / 0.07 ml (nAMD, DMO, macular oedema secondary to RVO) 0.4 mg / 0.01 ml^c^ (ROP)	6 mg / 0.05 ml (all indications)	6 mg / 0.05 ml (all indications)	1.25 mg / 0.05 ml
**Posology**^**b**^	**0.5 mg dose:** One injection every 4 weeks until maximum visual acuity and/or no signs of disease acitivity. Thereafter, a treat-and-extend regimen may be used to extend intervals (intervals should be extended at a maximum of 2-weekly increments for nAMD and 4-weekly increments for DMO). No maximum interval is specified. **0.2 mg dose:** One injection per eye; up to three injections per eye may be administered within 6 months of starting treatment.	**2 mg dose:** One injection every 4 weeks for the first three doses in nAMD and for the first five doses in DMO, followed by one injection every 8 weeks; in RVO, injections should be given every 4 weeks until maximum visual acuity and/or no sign of disease activity. Thereafter, a treat-and-extend regimen may be used to extend intervals at 2- or 4-weekly increments. No maximum interval is specified. For mCNV, a single injection is required. **8 mg dose:** For treatment-naïve patients, one injection every 4 weeks for the first three doses in nAMD, DMO and RVO. Then, in nAMD and DMO, intervals may be extended up to every 4 months and further extended using a treat-and-extend regimen. Patients can be extended to a maximum interval of 24 weeks. In RVO, injection intervals may be extended after the first three doses according to the physician’s judgement, and no maximum interval is specified. For patients who are switching from another anti-VEGF treatment and have stable visual and/or anatomic outcomes, previous treatment intervals can be maintained or extended after the first injection of aflibercept. For those switching and have suboptimal visual and/or anatomic outcomes, aflibercept may be initiated with one every injection every 4 weeks for the first three doses, followed by a treat-and-extend regimen. A treatment interval should be no shorter than 4 weeks. **0.4 mg dose**: One injection per eye for the treatment of ROP; up to two injections per eye may be administered within 6 months of starting treatment.	One injection every 4 weeks for the first three doses in nAMD and one injection every 6 weeks for the first five doses in DMO; thereafter, the treatment interval may be extended or shortened by increments of 4 weeks (based on disease activity). No maximum interval is specified for either indication.	One injection every 4 weeks for the first three doses for all indications. Thereafter treatment is individualised; in nAMD, a maximum interval of 16 weeks in patients without disease activity should be considered and in those with disease activity, treatment every 8 or 12 weeks should be considered. In DMO and macular oedema secondary to RVO, a treat-and-extend regimen may be used to extend intervals at 4-weekly increments; no maximum interval is specified.	One injection every 4 weeks until maximum visual acuity and/or no signs of disease acitivity. The kinetics of bevacizumab gamma efficacy suggest that three or more initial consecutive monthly injections may be needed. Thereafter, the treatment intervals can be individualised at the healthcare practitioner’s discretion. No maximum interval is specified.
**Longest studied interval between injections in pivotal trials**^**d**^	12 weeks (nAMD) and 12 weeks (DMO)	**2 mg dose:** 16 weeks (nAMD), no upper limit (DMO) 8 weeks (RVO) [9] **8 mg dose:** 24 weeks (nAMD and DMO) and 16 weeks (RVO)	20 weeks (nAMD) and 16 weeks (DMO)	16 weeks (nAMD, DMO and RVO)	1 month

*AP-ROP* aggressive posterior retinopathy of prematurity, *CNV* choroidal neovascularisation, *DMO* diabetic macular oedema, *IVT* intravitreal, *mCNV* myopic choroidal neovascularisation, *nAMD* neovascular age-related macular degeneration, *PDR* proliferative diabetic retinopathy, *ROP* retinopathy of prematurity, *RVO* retinal vein occlusion, *VEGF* vascular endothelial growth factor.

^a^Molecular structures are visuals for illustration purposes only and are not drawn to scale.

^b^According to the summary of product characteristics published by the European Medicines Agency or European Commission correct as of February 2026.

^c^Indicated in pre-term infants only.

^d^Ongoing clinical trials for various IVT anti-VEGF agents are currently underway; pending clinical trial results, the duration of these intervals for various indications may change in the future.

Although some patients can achieve extended treatment intervals with IVT anti-VEGF therapies beyond the standard intervals described in the prescribing information of the respective product in clinical practice (see Table 1), there are also patients who cannot maintain their vision improvements with extended treatment intervals and require more intensive treatment [16–18]. Such patients may be undertreated if treated using extended intervals, and would therefore be at risk of losing vision. This variability could be due to patient or disease characteristics and is affected by the pharmacological properties of the drug being used. Regardless of the cause, there is a need to improve the durability profiles of current IVT therapies in all patients.

The durability of a drug refers to its ability to delay the progression of disease, in a manner that is well tolerated by the patient [19]. For most treating physicians, this is interpreted as how long the drug has the desired effect. Despite differences in underlying disease processes, the pathogenesis of nAMD and DMO are both primarily driven by increased levels of VEGF, with other molecular drivers such as angiopoietin-2, and inflammatory molecules such as placental growth factor, platelet-derived growth factor, IL-6, IL-1ß, TNF-α and intracellular adhesion molecule 1 also considered relevant, especially in the pathogenesis of DMO [20–23]. Emerging therapies are looking into additional targets beyond VEGF; however, improving the durability profiles of current anti-VEGF therapies could therefore be achieved by increasing the time VEGF is suppressed in the eye, which would reduce the number of injections needed and result in longer intervals between each injection.

In addition to various patient factors, the duration of the therapeutic effect of a drug is determined by several pharmacological properties. This article will review key pharmacological principles concerning approved IVT anti-VEGF treatments for nAMD and DMO and aims to elucidate their meaning and relevance for effective pharmacological management of retinal diseases. These properties can influence key outcomes, such as the duration of the therapeutic effect, and are relevant to the needs of ophthalmology clinical practice.

## Key pharmacological terms

Pharmacology is the study of the effect of drugs on living systems and their interactions with these systems [24]. In very general terms, pharmacology can be divided into pharmacokinetics and pharmacodynamics [24]. Pharmacokinetics refers to the absorption, distribution, metabolism and excretion (ADME) of drugs, i.e., the effect the body has on the drug [24]. Pharmacodynamics refers to the biological effect of a drug and its mechanism of action, i.e., the effect the drug has on the body [24].

The pharmacodynamic effects of drugs are mediated through drug–receptor interactions (or in the case of IVT anti-VEGF therapies, drug–ligand interactions) and the subsequent modulation of the downstream cellular signalling pathway [25]. When drugs bind to their corresponding receptors or ligands at a binding site to form drug–receptor or drug–ligand complexes, they behave as an agonist or an antagonist. Agonists induce a physiological response similar to that caused by an endogenous ligand, whereas antagonists inhibit the physiological response, typically by preventing the endogenous ligand from interacting with the binding site [24, 26].

Understanding key pharmacokinetic and pharmacodynamic principles can help clinicians fully understand the differences between various therapies, so that the best choice of treatment and regimen can be made for the patient. The following are pharmacological terms or parameters that influence the clinical outcomes of a drug, such as the duration of the therapeutic effect. They will be discussed in detail here, but the terms are also summarised in Table 2.

**Table 2:** Glossary of key pharmacological principles.

Key term	Definition
Dose	Mass of a drug administered at one time
Mole	Unit of measurement that expresses the amount of active substance in a sample (one mole of a substance contains the same number of molecules as one mole of another substance with a different molecular weight)
Molecular weight	Overall mass of a molecule
Molar dose	Number of moles in a dose
Concentration	Amount (typically mass) of active substance in a specified volume
Molar concentration	Amount of moles of an active substance in a specified volume
Clearance	The volume of plasma that is completely cleared of a drug in a specific amount of time
Ocular clearance^a^	The theoretical volume of the eye that is completly cleared of a drug in a specific amount of time
Volume of distribution	The apparent volume into which a drug distributes in the body at equilibrium based on bioavailable drug dose and drug concentration in plasma
Ocular volume of distribution^a^	The assumed volume into which a drug distributes within the eye to estimate a concentration in the eye based on drug dose
Half-life	Time for the concentration or amount of a drug to fall by half
Efficacy	Measure of the response to a drug
Binding affinity K_D_	Strength of the interaction between the drug and its target Equilibrium dissociation constant, used to quantify the strength of the interaction
Potency EC_50_ IC_50_ ED_50_	Concentration or dose of a drug needed to elicit a response of a certain magnitude Concentration of drug required to **produce** half of the maximum response Concentration of drug required to **inhibit** half of the maximum response Dose of drug required to produce half of the maximum response

^a^In the eye.

### Chemical properties of a drug (active ingredient of a medicine)

#### Mole

A mole is a unit of measurement that expresses the amount of a substance and is denoted using the abbreviation ‘mol’. One mole always contains 6.02214076 × 10^23^ (also referred to as Avogadro’s number) elementary entities (e.g., molecules), irrespective of the substance or its molecular weight. This allows head-to-head pharmacological comparison of the number of molecules in different treatments. For example, 1 mol of ranibizumab, 1 mol of brolucizumab and 1 mol of aflibercept contain the same number of molecules of active substance, despite having very different molecular weights and pharmacological properties.

#### Molecular weight / molar mass

In chemical terms, the molecular weight of a drug is the sum of the weight of the atoms in one molecule of that drug (i.e., the overall mass per molecule) and is expressed in Daltons (Da). The molecular weight of a small molecule drug is typically <1 kDa; an example is acetazolamide, which is used to treat glaucoma, among other diseases, and weighs 222 Da [27, 28]. Conversely, the molecular weight of biologics can reach up to 1 000 kDa. For example, the molecular weights of anti-VEGF therapies such as brolucizumab, ranibizumab, aflibercept and faricimab are 26 kDa, 48 kDa, 115 kDa and 150 kDa, respectively [29, 30]. Molecular weight (or effective molecular size) inversely relates to diffusion. Diffusion in the vitreous is generally an important determinant for ocular clearance; larger drugs generally diffuse and clear more slowly than smaller ones [31]. Closely linked to the molecular weight is the molar mass of a substance, i.e., the mass per 1 mol, expressed as g/mol. For example, acetazolamide has a molar mass of 222 g/mol [32].

### Pharmacological properties of a drug (active ingredient of a medicine)

#### Binding affinity

**Fig. 1:**
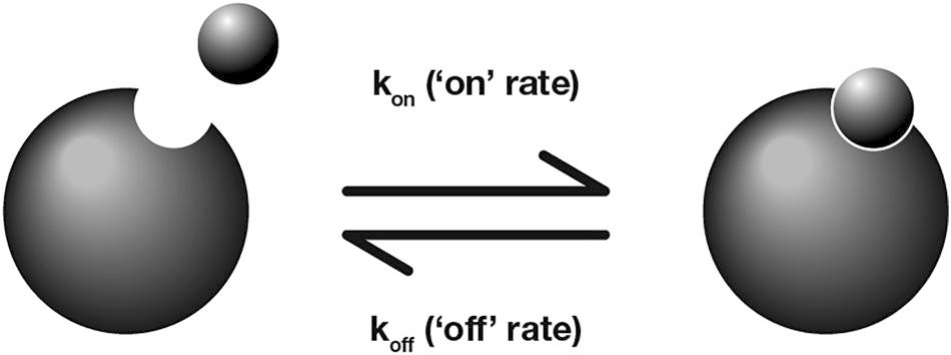
The relationship between ‘on rate’ and ‘off rate’ during dynamic equilibrium. k_off_ is the rate constant of dissociation of the drug from its target, whereas k_on_ is the rate constant of association of the drug to its target. The dissociation constant (K_D_) is the ratio of the k_off_ and the k_on_. The lower the K_D_ of a drug, the harder it is for the drug to dissociate from its target, meaning that such a drug would have a high binding affinity to its target.

In pharmacological terms, the binding affinity describes the strength of the interaction between the drug and its target (be that a receptor or a ligand) [33, 34]. When drug molecules bind to their target, a dynamic equilibrium of binding to and dissociation from the target is established. The rate at which the drug binds to the target is referred to as the ‘on rate’ (k_on_), and the rate at which the drug dissociates from the target is referred to as the ‘off rate’ (k_off_; Fig. 1). The tendency of the drug and its target to dissociate from one another is described using the equilibrium dissociation constant (K_D_; i.e., the ratio of the off and the on rate); this term is also used to denote the binding affinity of a drug [33].

$${{\rm{K}}}_{{{\rm{D}}}} = \frac {{{\rm{k}}}_{{{\rm{off}}}}} {{{\rm{k}}}_{{{\rm{on}}}}}$$

The lower the K_D_, the stronger the interaction between the drug and its target, and thus the greater the number of receptors occupied by the drug at any given time and concentration; therefore, a low K_D_ indicates a high binding affinity.

#### Efficacy

**Fig. 2:**
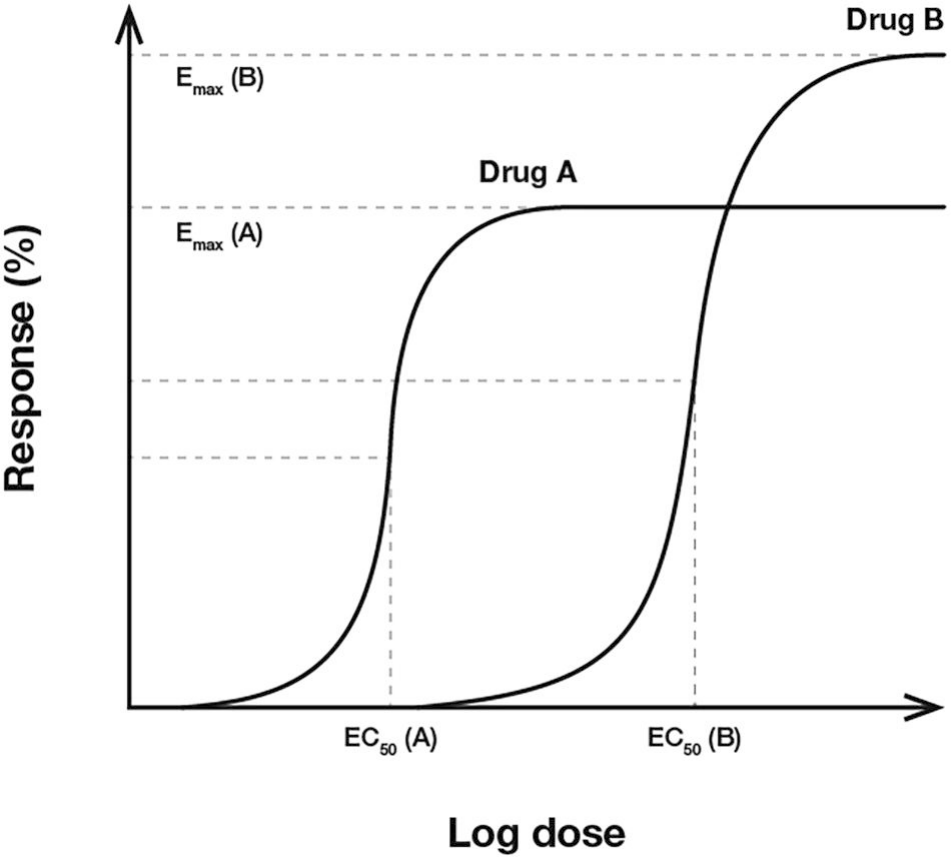
Dose–response curves depicting the different efficacy profiles and potency of different agonists. Drug A is the most potent agonist, followed by Drug B, as a lower dose of Drug A is required to induce half of its maximum response (hence, Drug A has a lower EC_50_). Drug B has a higher efficacy (and therefore a higher E_max_) than Drug A, as Drug B can induce a response of a higher magnitude compared with Drug A. *EC*_*50*_ half maximal effective concentration, *E*_*max*_ maximum effect.

In pharmacological terms, efficacy is the measure of the response of a drug [25]. The maximum response of a drug is typically represented by the E_max_ (see Fig. 2). Agonists have variable degrees of efficacy; a full agonist can produce the maximum possible response at the target, whereas a partial agonist produces less than the maximum possible response [24]. In antagonistic drug–receptor interactions, antagonists have affinity for their receptor but do not modulate the target themselves; that is, they are able to bind to their receptor but do not elicit a response. However, with their binding to the target, they prevent the binding of endogenous agonists to the receptor and thus prevent a downstream signalling cascade [24, 35]. During drug–ligand interactions, antagonists can inhibit agonists from binding to their receptor to produce a response [24, 35].

It should be noted that the efficacy of a drug as described previously is not synonymous with its effectiveness. The effectiveness of a drug refers to its ability to produce a desired therapeutic response, although this is often referred to as clinical efficacy.

#### Potency

Potency describes the relationship between the concentration of the drug and the magnitude of its effect [24, 25]. The terms ‘potency’ and ‘efficacy’ are often (and incorrectly) used interchangeably. Efficacy is the ability of a drug to produce a maximum response, whereas potency refers to the concentration of the drug necessary to achieve a response of a certain magnitude, e.g., the half-maximum response.

In the hypothetical scenario in Fig. 2, Drug A is more potent than Drug B; that is, a lower concentration of Drug A is required to elicit a half-maximum response compared with Drug B. However, at higher concentrations, Drug B is able to produce a response of greater magnitude compared with Drug A and consequently has greater efficacy (E_max_) than Drug A.

**Fig. 3:**
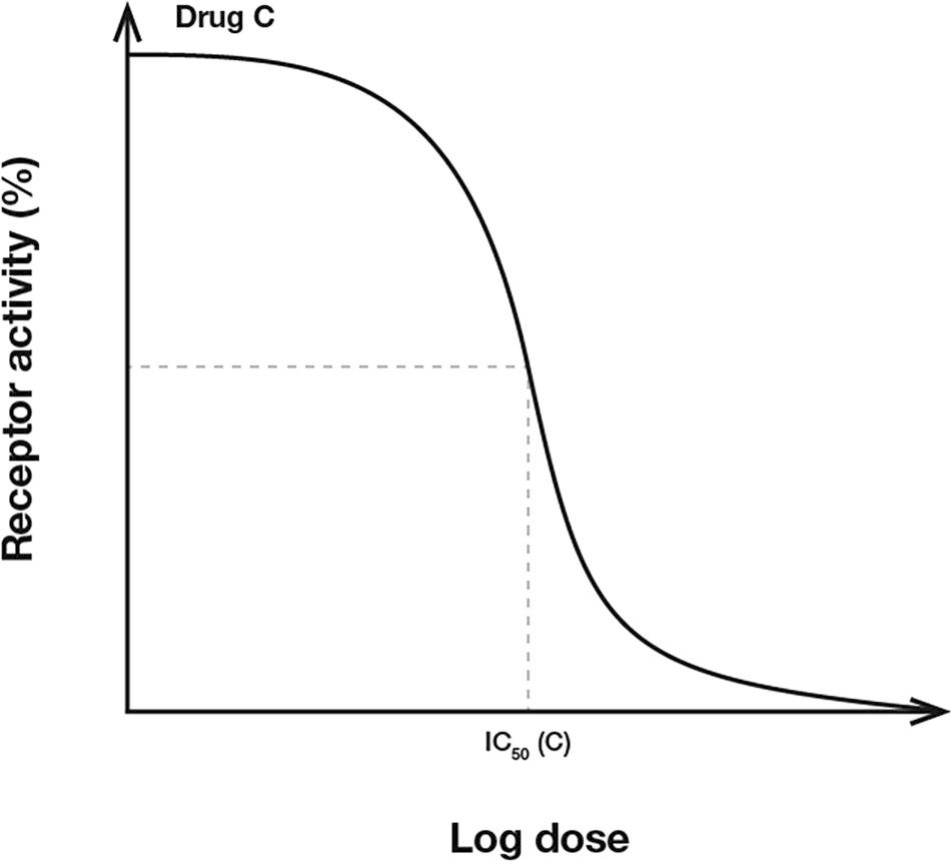
Receptor activity profile of an antagonist. Increasing the dose of Drug C, an antagonist, diminishes the maximum response seen at its receptor. The IC_50_ can be calculated when half of the maximum response has been inhibited. *IC*_*50*_ half maximal inhibitory concentration.

The parameter traditionally used for measuring the potency of an agonist is referred to as the EC_50_, which is defined as the concentration of drug required to produce half of the maximum response [25]. Potent agonists will induce a greater response with a lower concentration; therefore, the lower the EC_50_ value, the more potent the agonist (see Fig. 2). The potency of an antagonist may be described by the IC_50_, which is defined as the concentration of drug required to inhibit half of the maximum response (see Fig. 3) [36]. Potent antagonists will induce a greater inhibitory effect with a lower dose; therefore, the lower the IC_50_ value, the more potent the antagonist.

### Therapeutic interventions

#### Dose

A dose is the amount of drug administered at one time and is usually measured in units of mass (such as g or mg). The dose of a drug may differ based on the drug used, or the indication see (Table 1).

#### Molar dose

The dose can also be expressed as the number of moles of a drug taken, which is referred to as the molar dose. The relationship between dose, molar dose and molecular weight is defined as:

$${{\rm{Molar}}}\, {{\rm{dose}}} = \frac{{{\rm{Dose}}}} {{{\rm{Molecular}}}\, {{\rm{weight}}}} $$

Given that there are different molecular weights for different drug substances, molar doses allow a more meaningful comparison across different compounds binding to the same target. For example, the molecular weights of the anti-VEGF therapies brolucizumab, ranibizumab and aflibercept are 26 kDa, 48 kDa and 115 kDa, respectively. Therefore, on a molar basis, 6 mg of brolucizumab equals approximately 12 times the 2 mg dose of aflibercept and approximately 22 times the 0.5 mg dose of ranibizumab [29].

#### Additional points for consideration

When comparing equal molar doses of different anti-VEGF compounds, the number of ligand binding sites per anti-VEGF molecule is a factor to be considered. For instance, VEGF binds aflibercept in a 1:1 stoichiometry, i.e., each aflibercept molecule has one VEGF binding site [37]. In contrast, ranibizumab has a 1:2 ratio which means that two ranibizumab molecules bind to one VEGF molecule; bevacizumab has a 2:1 ratio which results in two VEGF binding sites per molecule [37]. However, to establish the pharmacological relevance of this factor, the binding affinity, potency and pharmacological efficacy of the drug must also be considered.

### Pharmacokinetics

#### Concentration

The concentration of a drug is defined as the amount of active substance in a certain volume. It can be expressed as the mass of drug in a specified volume (g/l or mg/ml) or as the number of moles in a specified volume (mol/l); the latter is also referred to as the molar concentration.

#### Bioavailability

The absolute bioavailability is the fraction of the administered dose (usually in %) reaching the systemic circulation. For systemically acting drugs, a high bioavailability is desirable. Bioavailability has less relevance for drugs that are locally administered, such as IVT anti-VEGF agents.

#### Volume of distribution

The volume of distribution describes the apparent volume into which a drug distributes in the body at equilibrium and reflects the tendency of a drug to distribute to compartments or tissues outside the systemic circulation. It is the ratio of the bioavailable dose and the concentration in the circulation (plasma).

#### Clearance

Drug elimination is the process by which a drug is removed from the body, and the rate of this elimination process is described by the clearance. Clearance is measured in units of volume in a specific amount of time (e.g., l/h) and reflects the theoretical volume of plasma completely cleared of the drug per unit of time.

In the context of intravitreally administered drugs, sometimes the term ‘ocular clearance’ is used and refers to the process by which the drug is removed from the eye. In fact, this is an absorption/distribution process from the site of injection (the vitreous) into the systemic circulation, where the drug is then completely cleared (i.e., eliminated from the body).

#### Half-life

The half-life of a drug usually refers to the elimination half-life; that is, how long it takes for the amount of drug in the body (or at its site of action) to reduce by half. For directly acting and not covalently binding agonists or competitive antagonists, this means that a drug with a long half-life will remain at its site of action and continue to have a biological effect for longer compared with a drug that has a shorter half-life. The half-life of a drug is a property dependent on the clearance and volume of distribution of a drug:

$${{\rm{Half}}}{\mbox{-}}{{\rm{life}}} \, ({{\rm{h}}}) = 0.693 \times \frac{{{\rm{Volume}}}\, {{\rm{of}}}\, {{\rm{distribution}}}\, ({{\rm{l}}})} {{{\rm{Clearance}}} \,({{\rm{l}}}/{{\rm{h}}})}$$

For most drugs, the rate at which the drug is absorbed into the systemic circulation is quicker than the rate at which it is eliminated from the circulation [38]. Hence, the terminal half-life is determined by the rate of elimination. However, for some IVT anti-VEGF therapies, such as aflibercept, the rate at which they are absorbed (or cleared) from the vitreous into the systemic circulation is much slower than the rate at which they are eliminated from the systemic circulation [38, 39]. This phenomenon is known as ‘flip-flop kinetics’ [39]. The vitreous behaves as a depot, slowly releasing the drug into the systemic circulation, and that rate of release from the eye (i.e., ‘ocular clearance’) determines the apparent half-life in plasma [38].

## Discussion

The relationship between the different pharmacokinetic and pharmacodynamic parameters for a drug is often complex. There is significant interplay between some of the parameters discussed previously, which can influence the therapeutic effect of a drug [40]. For instance, the high potency of a drug with a non-specific target binding profile could result in a suboptimal safety profile, as the drug could yield a number of unwanted ocular or systemic effects; and a drug with a high affinity is desired when the drug needs to be long-acting, but such a drug may not be best suited in scenarios where its action needs to be short-lived. Furthermore, various individual patient factors can also change the pharmacological profile of a drug and hence alter its therapeutic effects.

A clinical scenario where the pharmacological parameters of an anti-VEGF agent has potential practical relevance may be in patients with a high treatment demand. For example, disease activity recurrence within a short time after treatment may indicate a high VEGF load driving the disease pathology, and necessitate frequent injections. In such cases, the treating ophthalmologist may want to consider switching the current agent for an option with either a: higher dose; greater target potency; longer half-life; or slower ocular clearance, in an attempt to increase the time VEGF is suppressed in the eye, to maintain longer disease control and hence potentially result in longer intervals between each injection.

The goal for IVT therapies is to achieve efficacy without safety concerns, while minimising the number of IVT injections needed to manage disease and helping to minimise treatment burden. In the following sections, the pharmacological properties of currently available IVT anti-VEGF therapies are discussed in the context of optimal disease management.

### How do the pharmacological properties of IVT anti-VEGF therapies affect the number of IVT anti-VEGF injections needed to manage retinal disease?

Anti-VEGF therapies act as antagonists, as they bind to their respective VEGF ligands and prevent them from binding and activating VEGF receptors [41].

After an anti-VEGF is injected, their size (i.e., their molecular weight) is a property that influences how quickly they are distributed through and cleared from the eye, which therefore influences their ocular half-life [40, 42]. Drug elimination through the anterior chamber is based on diffusion through the vitreous humour, eventually reaching the aqueous humour [40]. Upon reaching the aqueous humour, the drug is eliminated in its outflow [40]. Given their size, large drugs (i.e., those with a high molecular weight) such as IVT anti-VEGF therapies take longer to diffuse through the vitreous compared with smaller drugs [31, 40, 43]. Moreover, ocular barriers such as the blood–aqueous and blood–retinal barriers do not allow large molecules and proteins to pass through; however, they do allow the permeation of small molecules [40]. Smaller molecules are mainly cleared via a posterior route in the back of the eye given their ability to cross these barriers [40, 44]. As a result of these factors, the ocular clearance of small molecules is much faster than the ocular clearance of large drugs, such as anti-VEGF therapies. This also means that the ocular half-lives of larger molecules like anti-VEGF therapies can be up to several days, whereas the half-lives of small molecules in the vitreous are typically only hours long [40]. Moreover, the addition of a neonatal Fc receptor (FcRn) portion on specific anti-VEGF molecules contributes towards a longer systemic half-life as it prevents lysosomal degradation of the drug and is involved with the recycling of monoclonal antibodies [40]. A study has also suggested that it may contribute towards the recycling of anti-VEGF therapies containing an FcRn portion in the eye [45].

Additionally, IVT anti-VEGF therapies used clinically often have strong binding affinities for VEGF-A; in a head-to-head comparison in a preclinical model, the reported K_D_ of aflibercept, ranibizumab and brolucizumab for VEGF-A were 0.1719 pM, 1.3 pM and 21.8 pM, respectively (note that a low K_D_ is indicative of a drug with a high binding affinity) [46]. The mean reported VEGF suppression time (i.e., the duration for which VEGF-A concentrations in aqueous humour are suppressed below the concentration required for therapeutic effect) for aflibercept and ranibizumab is 67–71 days and 34–38 days, respectively [47–49]. These VEGF suppression times may be a result of the long ocular half-lives (and thus, slower ocular clearance) and high binding affinity of these IVT anti-VEGF therapies and mean that such therapies may be administered at treatment intervals of several weeks or months.

Moreover, current IVT anti-VEGF therapies are potent compounds acting at low concentrations; in a head-to-head comparison in a preclinical model, the reported IC_50_ concentrations of aflibercept, ranibizumab and brolucizumab for VEGF-A were 2.42 nM, 10.82 nM and 5.74 nM, respectively (note that the lower the IC_50_ value, the more potent the drug) [46]. Given that a therapy with higher potency can inhibit its target at lower concentrations, potent anti-VEGF therapies have the potential to be more durable than less potent therapies when administered at long dosing intervals, assuming otherwise identical pharmacokinetic behaviour, molar doses and pharmacodynamics.

**Fig. 4:**
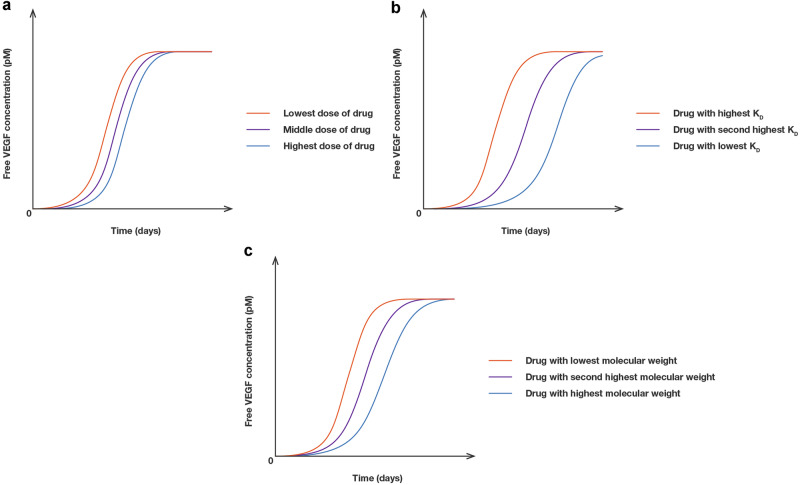
Simulated effect of adjusting various parameters of an anti-VEGF drug on the free VEGF concentration in the aqueous compartment of patients with nAMD [50]. **a**.** Simulated effect of increasing dose**. **b Simulated effect of increasing binding affinity (lowering K**_**D**_**)**.** c Simulated effect of increasing molecular weight.** Reprinted (adapted) with permission from Hutton-Smith LA, et al. Mol Pharm. 2016;13:2941–2950. Copyright 2016 American Chemical Society. **a** Simulated effect of increasing the dose: orange represents the lowest dose, purple represents the next highest dose, and blue represents the highest dose. **b** Simulated effect of lowering the K_D_ (with a constant half-life): orange represents the highest K_D_, purple represents a lower K_D_, and blue represents the lowest K_D_. **c** Simulated effect of increasing the molecular weight:^a^ orange represents the lowest molecular weight, purple represents the next highest molecular weight, and blue represents the highest molecular weight. ^a^The mass dose of the drug was adjusted in proportion to the molecular weight, keeping the molar dose constant. *K*_*D*_ equilibrium dissociation constant, *nAMD* neovascular age-related macular degeneration, *VEGF* vascular endothelial growth factor.

Despite these favourable properties, there remains an unmet need to further reduce treatment burden with IVT anti-VEGF therapies. Adjusting the pharmacokinetic and pharmacodynamic properties of currently existing anti-VEGF therapies could potentially improve the durability profiles of IVT anti-VEGF therapies while maintaining their efficacy profiles. In a theoretical, mechanistic model of the IVT pharmacokinetics of anti-VEGF therapies, increasing the molar dose, increasing the binding affinity or increasing the molecular weight of anti-VEGF therapies theoretically increased the duration of VEGF suppression and potentially the durability of such drugs (Fig. 4) [50]. In Fig. 4a−c, Day 0 represents the point of maximum VEGF suppression by the anti-VEGF therapy and the y-axis represents the concentration of free VEGF, which increases when fewer VEGF molecules are bound by anti-VEGF treatment and VEGF suppression decreases; the highest point on the graph is the point where no VEGF is suppressed and the free VEGF concentration is high. Thus, a curve shifted to the right represents a longer duration of VEGF suppression. All three of the theoretical models shown in Fig. 4 assume that only one factor is changed (i.e., A: dose; B: binding affinity (K_D_); and C: molecular weight) while all other pharmacokinetic and pharmacodynamic properties remain the same. Figure 4a–c here are only intended to visualise the concept of the potential effect of such changes; they do not provide insights into the quantitative effect size, for which the original paper by Hutton-Smith et al. should be consulted [50]. As seen in Fig. 4a and b, a higher dose or a higher binding affinity (lower K_D_) are associated with an increased duration of VEGF suppression. Figure 4C demonstrates how the VEGF suppression time is also expected to increase when the molecular weight of a drug is increased (with the mass dose being adjusted in proportion to the increase in molecular weight, i.e., keeping the molar dose the same) [50]. However, the binding affinity and the molecular weight of a drug are properties that are inherent to the molecule and cannot be changed without altering the molecule itself. Conversely, the dose of a drug can be altered to influence its pharmacological profile and durability of effect in particular without making changes on a molecular level. The suitability of this concept has been shown clinically.

Clinical trials have shown that, compared with lower doses, higher doses of IVT anti-VEGF therapies were associated with the need for fewer injections in patients with nAMD or DMO while maintaining the clinical effectiveness [51]. In PULSAR (nAMD) and PHOTON (DMO), it was demonstrated that patients treated with aflibercept 2 mg (after three initial monthly doses in PULSAR and five initial monthly doses in PHOTON) and patients treated with aflibercept 8 mg (after three initial monthly doses in both studies) achieved comparable, non-inferior vision gains, but those treated with the higher dose received fewer injections per protocol. At the Week 48 time point in PULSAR and PHOTON, patients treated with aflibercept 8 mg every 12 or 16 weeks achieved and maintained vision gains with 5.0 − 6.1 injections, whereas patients treated with aflibercept 2 mg every 8 weeks received 6.9 − 7.9 injections [52, 53]. Although it was demonstrated in HARBOR (nAMD) that patients treated with ranibizumab 2 mg *pro re nata* (PRN) achieved and maintained similar vision gains over 2 years with fewer doses compared to patients treated with 0.5 mg PRN, the HARBOR study did not meet its primary endpoint, as there was no evidence that the 2.0 mg monthly regimen achieved superiority in vision gains compared with the 0.5 mg monthly regimen [54, 55]. Additionally, both PRN regimens failed to meet the non-inferiority margin when compared to the 0.5 mg monthly regimen.

Although it may be expected that higher doses of the same drug would result in fewer injections and increased durability, the anticipated extension of injection intervals does not necessarily extend proportionally. Based on a published model [39], increasing the dose of aflibercept from 2 mg to 8 mg while assuming same ocular clearance would theoretically extend the dosing interval from 8 to 11 weeks (i.e., provide an additional 3 weeks between treatment intervals). However, a population pharmacokinetic model and simulation that included data from the pivotal clinical trials for aflibercept 8 mg (PULSAR and PHOTON) surprisingly revealed that, compared with aflibercept 2 mg, aflibercept 8 mg suppressed VEGF for approximately 6 to 8.9 weeks longer due to not only a higher molar dose, but also a slower ocular clearance [56].

Whereas VEGF is considered the primary driver of disease pathology in nAMD and DMO and is the primary target for current therapies, other molecular targets may also be relevant. For example, faricimab is a bispecific antibody that binds both VEGF-A and angiopoietin-2, with the aim of increasing treatment durability [20]. Similarly, aflibercept targets VEGF-A, VEGF-B and placental growth factor, all of which are implicated in the pathogenesis of nAMD and DMO [20, 23, 57, 58].

Beyond IVT injections, there is also attention on novel delivery approaches for nAMD and DMO. These approaches include sustained delivery systems, such as biodegradable corticosteroid implants for DMO and reservoir systems, and long-acting gene therapies in various stages of development [59–62].

### How can patient factors affect pharmacological profiles?

The majority of pharmacokinetic and pharmacodynamic data are based on in vitro, in vivo and modelling data. However, there are clinical factors individual to the patient that may affect or alter the pharmacological profile of a drug in the eye; as such, the typical behaviour of the drug might deviate compared to its pharmacological profile. In some cases, patients may need their treatment regimens adapted to accommodate their clinical history. The examples discussed in the following section are general considerations and have been observed in different disease states using various types of ocular drugs. Please note that these observations should be treated with caution, as there is no existing clinical evidence to support how the various clinical factors discussed here may alter the pharmacological profile and effectiveness of IVT anti-VEGF drugs in clinical practice.

Preclinical models have suggested that anti-VEGF agents are cleared more rapidly in vitrectomised and/or lensectomised eyes compared with eyes that have not undergone such procedures [63–65]. The quality and the consistency of the vitreous humour could also potentially affect the pharmacokinetic profile of a drug. The viscosity of the vitreous humour decreases with age because of liquefaction [66], and this is caused by the degradation of the vitreous humour and affects the diffusion of drug molecules in the vitreous [44, 66]. A patient’s disease status could also potentially affect the pharmacological profiles of ocular drugs. A breakdown of the blood–retinal barrier due to various disease states could increase the elimination rate of IVT therapies from the vitreous [40]. Moreover, as shown in preclinical models treated with antibiotics, the presence of intraocular inflammation can also alter the intraocular half-life of a drug [67].

### Considerations for interpreting pharmacokinetic and pharmacodynamic studies

There are several considerations to make when interpreting pharmacokinetic and pharmacodynamic data. Caution should be exercised when comparing the pharmacological properties of different drugs, as different assays or experiments done under different conditions can yield different results [34]. Moreover, these assays may not reflect in vivo or clinical conditions. As such, direct comparisons between the pharmacological properties of different drugs can only be made when the assays used and the experimental conditions are identical [57]. Additionally, mathematical pharmacokinetic and pharmacodynamic models depend on the assumptions and data used to establish them, which has to be taken into consideration when comparing results.

Additionally, ocular pharmacokinetic profiles may vary between humans and other species, in part due to anatomical variation [40]. The most reliable pharmacokinetic and pharmacodynamic data are those obtained from analyses conducted in human clinical trials.

Finally, pharmacological and pharmacokinetic properties of a drug such as the binding affinity, molecular weight, potency and ocular half-life are all important factors contributing to its clinical effectiveness. However, none of these factors should be considered in isolation, and the complex interactions of the various factors need to be taken into account when discussing the clinical effectiveness and safety of IVT anti-VEGF therapies.

## Conclusions

Along with the mechanism of action, the pharmacokinetic and pharmacodynamic properties of ocular IVT therapies are important considerations influencing the therapeutic effect of a drug; where possible, consideration should be given to how these properties may affect clinical outcomes. However, it is worth considering that the clinical performance of a drug does not rely solely on its pharmacological profile but also depends on the interaction of these pharmacological properties with individual patient factors, which may vary widely.

Ultimately, the goal for any IVT anti-VEGF therapy used to treat nAMD or DMO is to suppress the target effectively and consistently, and preferably for the longest duration of time, to provide durable clinical outcomes and minimise the frequency of injections required. Despite IVT anti-VEGF therapies revolutionising treatment for nAMD and DMO over the last two decades and even with recent advancements enabling physicians to treat patients at extended treatment intervals, the treatment burden associated with these drugs remains a major unmet need. The pharmacokinetic and pharmacodynamic properties of a drug can inform the development of treatment options that are more durable than the current standard of care, to better meet the needs of patients.
